# Management of giant osteoma in the mandible associated with minor trauma: a case report

**DOI:** 10.1186/s13256-021-03217-2

**Published:** 2022-01-08

**Authors:** Isabela Wolf-Grotto, Lucas M. Nogueira, Basilio Milani, Erica C. Marchiori

**Affiliations:** 1 Division of Oral and Maxillofacil Surgery, Municipal Hospital Dr. Fernando Mauro Pires da Rocha, Estrada de Itapecerica, 1661, Vila Maracanã, São Paulo, Zip Code 05835-005 Brazil; 2 Residence Program of Municipal Hospital Dr. Fernando Mauro Pires da Rocha, São Paulo, Brazil; 3 Preceptor in Oral and Maxillofacial Surgery Residence Program of Municipal Hospital Dr, Fernando Mauro Pires da Rocha, São Paulo, Brazil

**Keywords:** Osteoma, Peripheral osteoma, Bone tumor, Bone pathology, Osteoma etiology, Case report

## Abstract

**Background:**

Osteoma is a benign tumor of the bones, which can be classified as central or peripheral. The occurrence in the jawbones is uncommon, but when it occurs, there is a greater prevalence of the mandible. The etiology is still unknown, and the hypothesis of its development is debated.

**Case presentation:**

A 35-year-old Caucasian man presenting a tumor lesion in the right jawbone that had been growing for 8 years sought medical service complaining of speaking impairment. According to the patient, the tumor appeared shortly after a minor trauma caused by tooth extraction. The diagnosis of the lesion was made through clinical, radiographic, and histological methods, and the surgical treatment was successful and satisfactory for the patient as well as the surgical team, despite a short follow-up.

**Conclusion:**

Etiopathogenesis of osteoma is not determined in the majority of cases. In the present report, it was possible to hypothesize the association between a minor trauma and the development of the tumor, reinforcing the reactive theory of tumor development. The uncommon location of the osteoma, as well the possibility of identifying the possible cause of the lesion, makes this case particularly interesting.

## Introduction

Osteoma is a benign, slow-growing, usually painless lesion characterized by the proliferation of compact or cancellous bone [1, 2]. Peripheral osteomas are usually found in the frontal, ethmoid, and maxillary sinuses, in which case they can sometimes cause headaches and sinusitis. The development of this tumor in the jawbones is uncommon, but when it occurs, there is a greater prevalence of the mandible [3–5].

Throughout the literature, we noticed variance on the matter of gender predilection. Most authors affirm that there is equal incidence among men and women [6, 7], while some claim to have found higher prevalence in men, up to a 2:1 ratio [4, 8, 9], and others in women, with results varying from a 2:1 to 1.5:1 ratio [3, 10, 11]. It has been claimed, even, that the real prevalence of osteomas is unknown [12], due to most lesions being asymptomatic and never reaching clinical proportions [10].

The etiology of peripheral osteoma is unclear, but there are currently three theories on its origin: developmental, neoplastic, and reactive to trauma [5, 13–15].

The purpose of this paper is to present the clinical, radiographic, and histopathological features, as well as the surgical treatment, of a peripheral osteoma in the mandible that has been directly associated with local minor trauma: a tooth extraction.

## Case report

A 35-year-old Caucasian male presented to the Maxillofacial Surgery service of Campo Limpo Hospital, in São Paulo, Brazil, with the main complaint of an intraoral swelling that had been growing for the last 8 years. The lesion was asymptomatic, and even though the patient had been aware of its constant growth, he decided to look for professional help only when its proportion began to cause him speaking impairment.

**Fig. 1.**
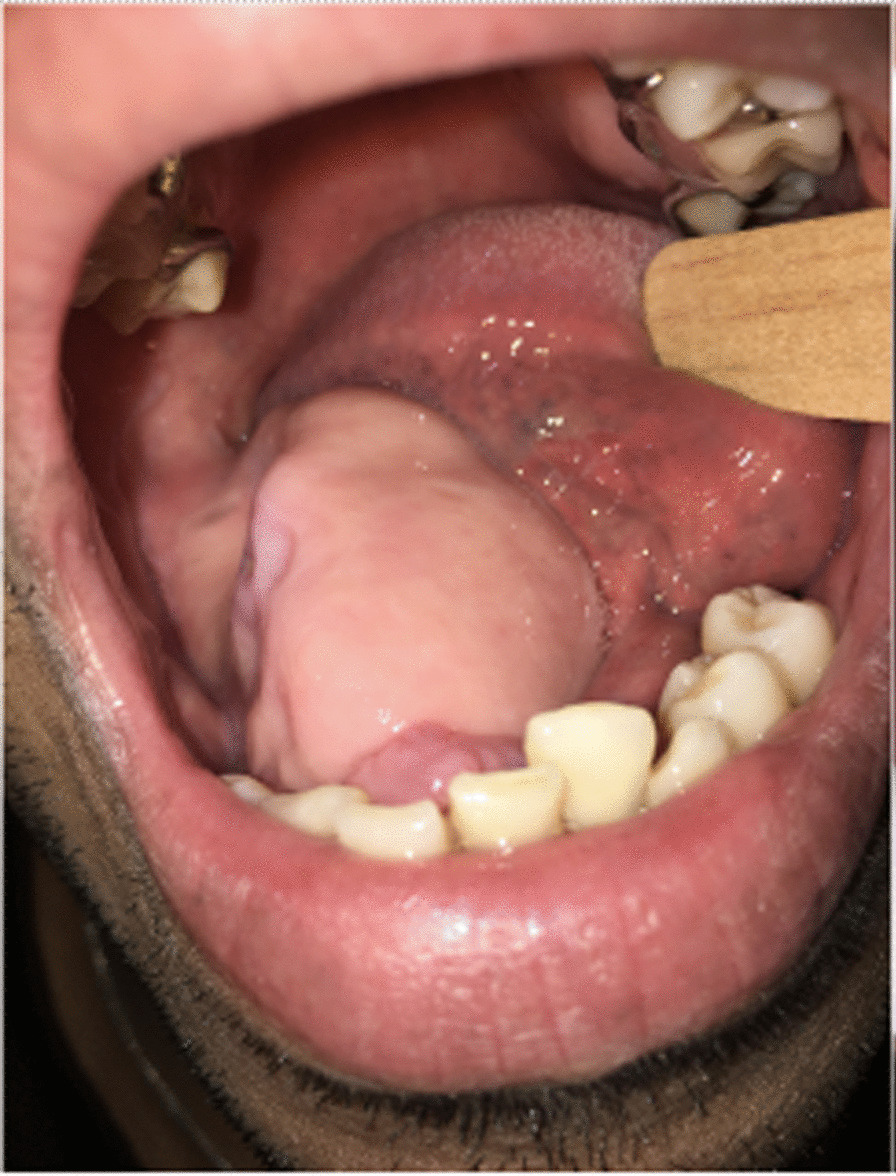
Clinical aspect of the lesion, showing the lesion in the inferior alveolar process of the mandible. The mas is covered by smoth, regular looking mucosa

**Fig. 2:**
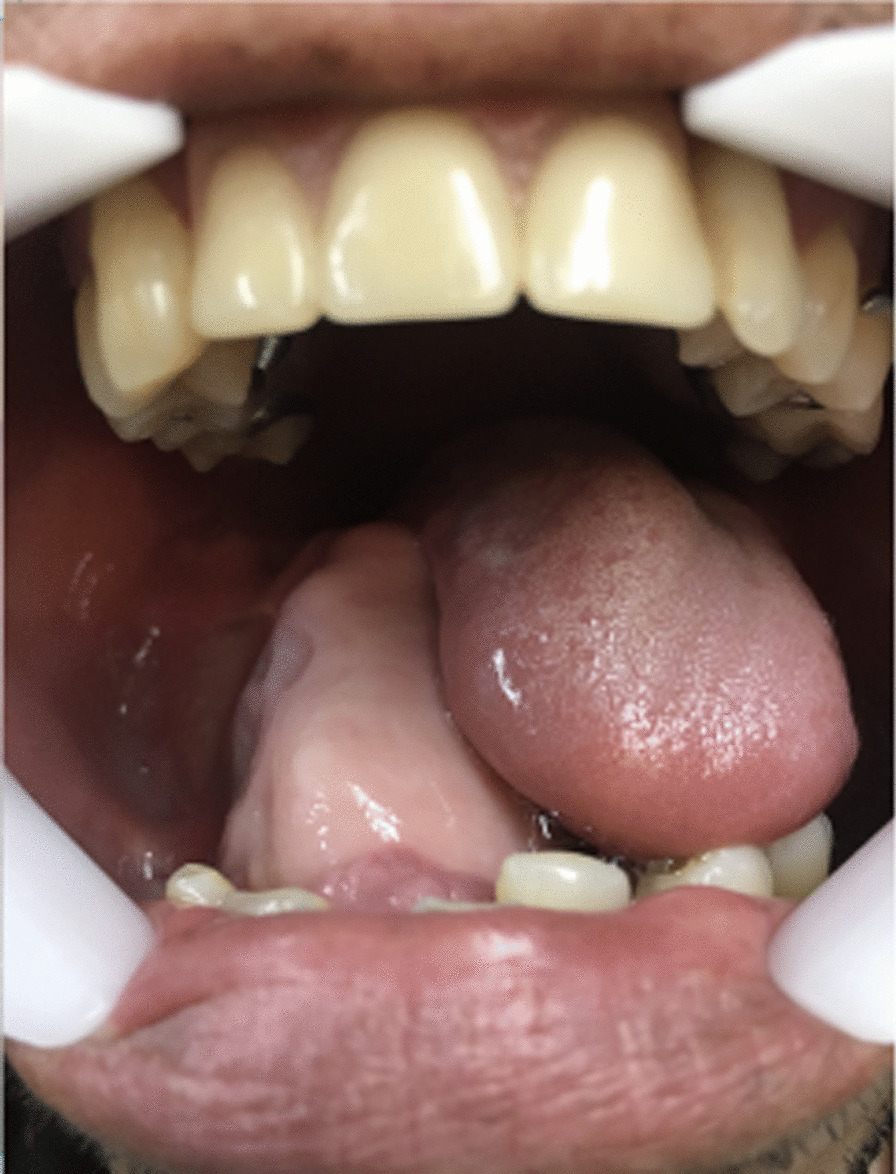
Clinical aspect of the lesion, demonstrating how such large proportion caused misplacement of the tongue while in resting position

The physical examination revealed a firm mushroom-like mass, with bony consistency covered by smooth regular mucosa spanning almost the entire right alveolar border of the mandible. The area was edentulous and, according to the patient’s report, had initiated its growth shortly after the extraction of a molar, which the patient could not specify. As it developed, the remaining teeth had been extruded until complete avulsion (teeth 28 through 32, in universal numbering system). No extraoral abnormalities were observed. The patient was in good health, with no history of previous diseases, smoking, or substance abuse. The diagnosis of Gardner syndrome was discarded due to the lack of any other symptoms, such as gastrointestinal implications or supernumerary teeth [16] (Figs. 1 and 2).

**Fig. 3.**
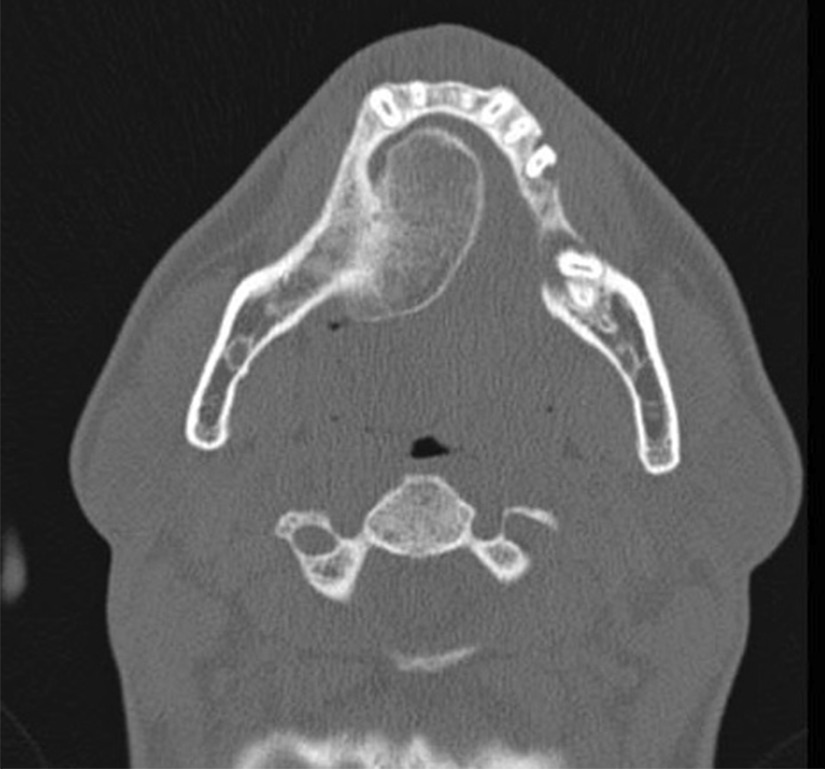
Computed tomography of the lesion in an axial cut, evidencing the mushroom-like shape attached to the alveolar process of the mandible and the radiographic characteristics of a normal bone, with central areal similar to medullary bone, surrounded by thin cortical bone

**Fig. 4.**
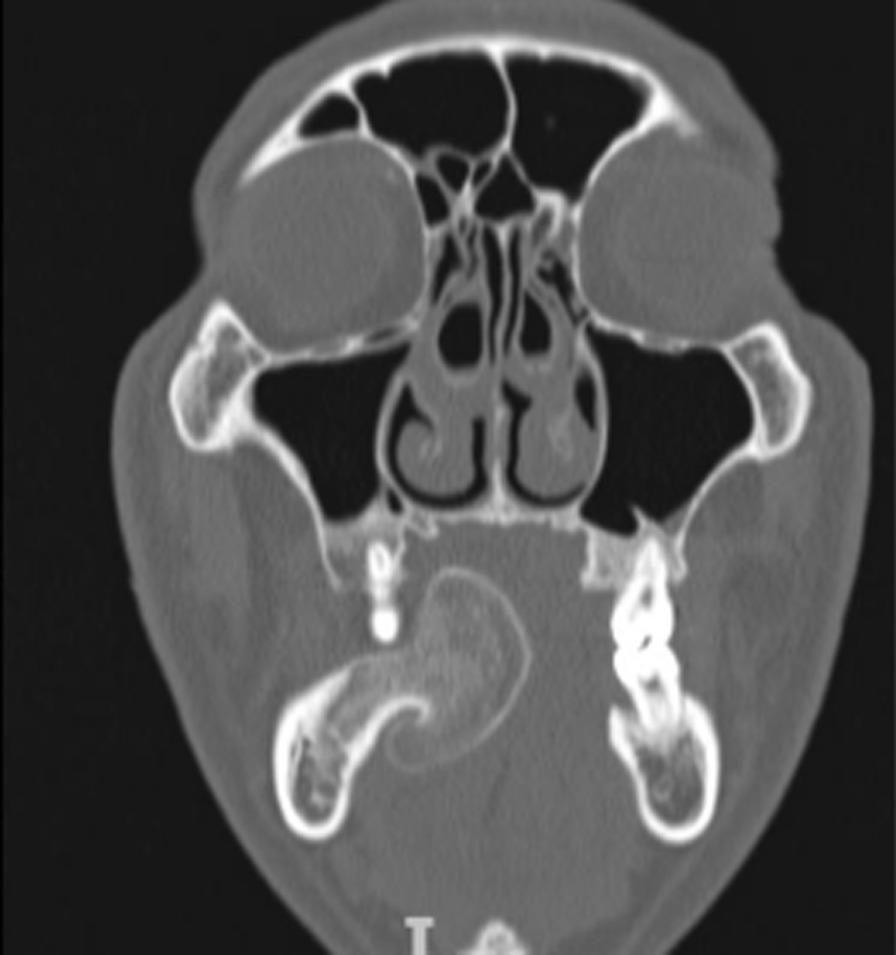
Coronal cut of the lesion in Computed Tomography image

Computed tomography revealed a well-defined pedunculated mass attached to the right body of the mandible with radiographic characteristics resembling the original bone, consisting of a central area of moderated radiopacity similar to medullary bone, surrounded by a denser, more radiopaque thin area, comparable to cortical bone. According to Rodriguez (2011), these particular growth characteristics make it easy to diagnose a peripheral osteoma clinically and radiographically (Figs. 3 and 4).

**Fig. 5:**
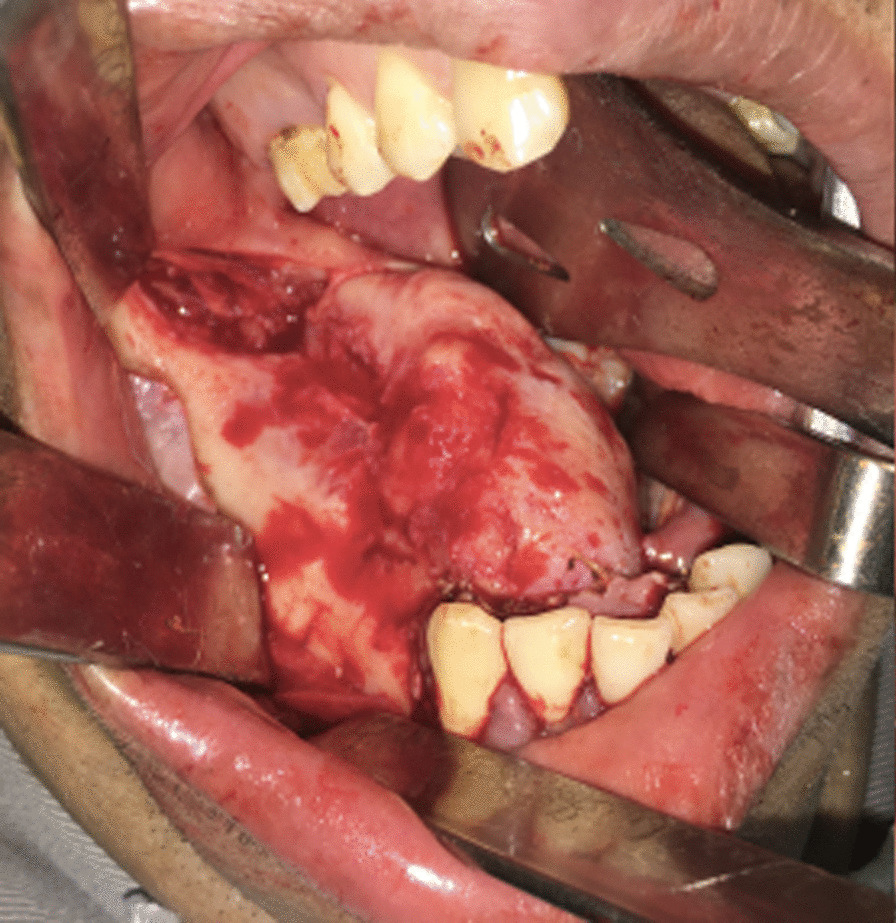
Aspect of the lesion during transoperative period, after divulsion of soft tissues and complete exposure of the lesion, which presented bony texture

**Fig. 6:**
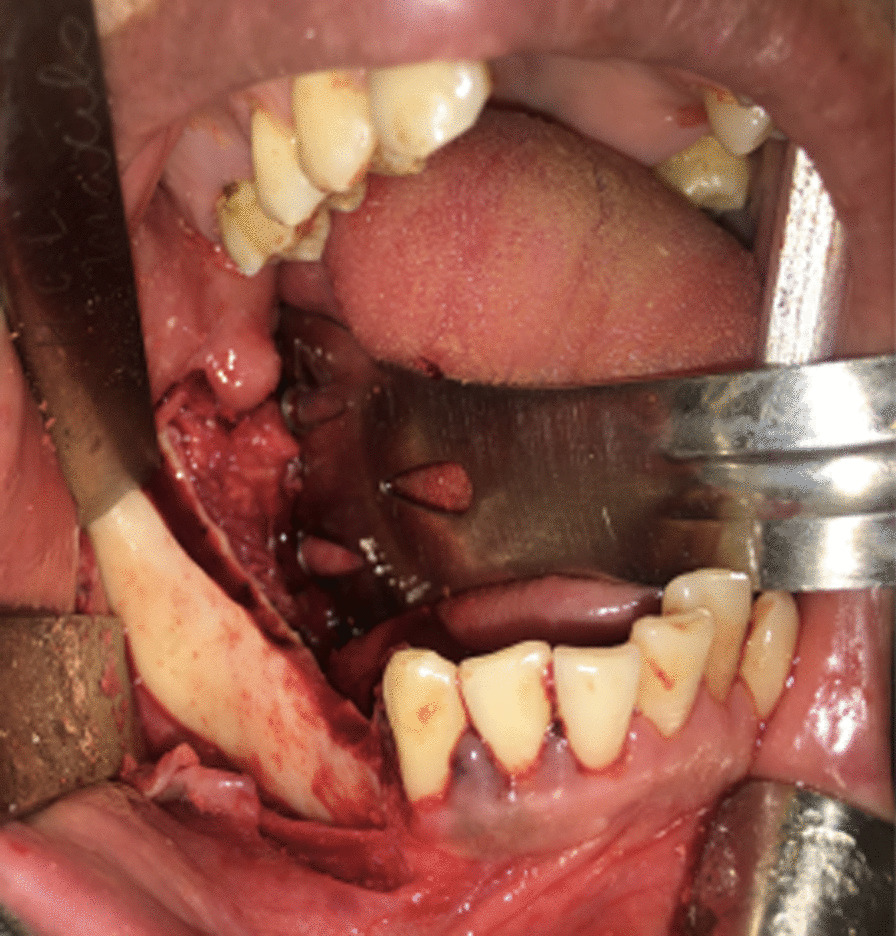
Transoperative image of the right alveolar process of the mandible after total excision of the mass and reshaping of the bone

**Fig. 7:**
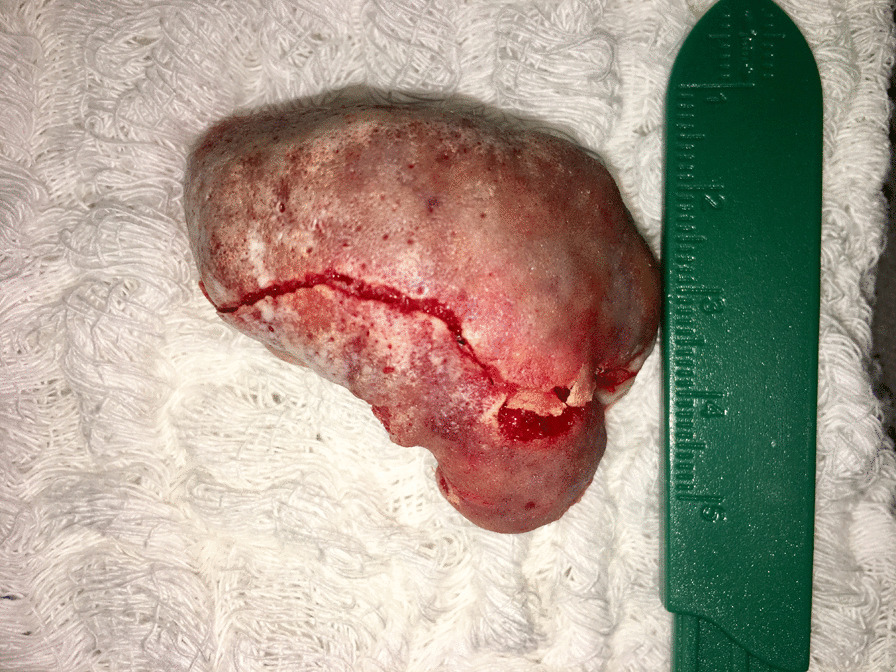
The excised piece next to a sizing reference (each line is 1 mm)

**Fig. 8:**
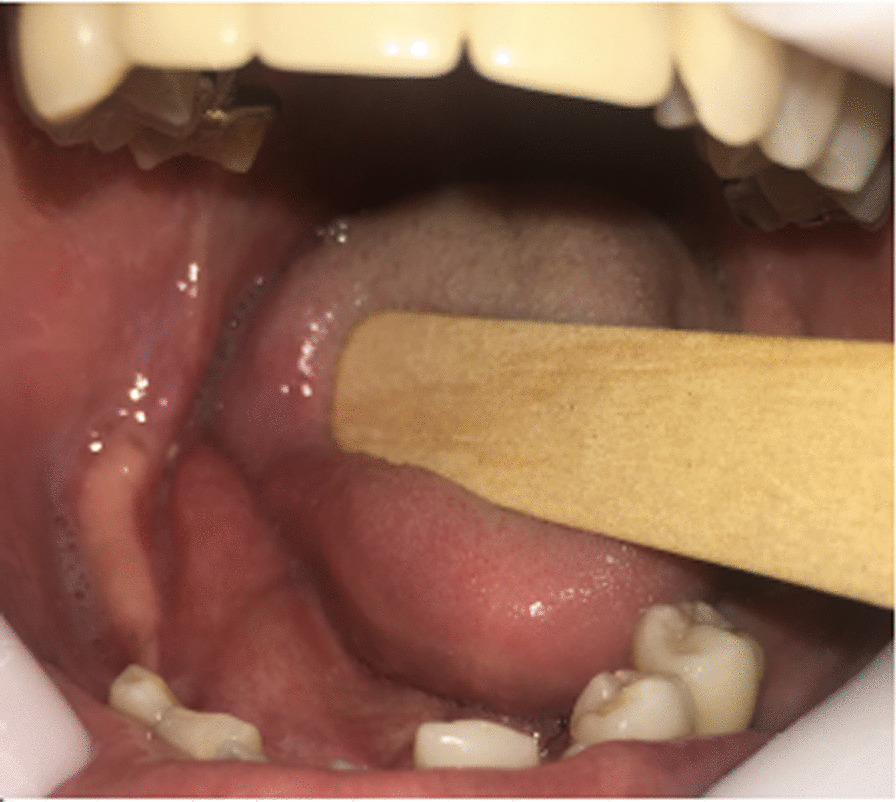
Clinical aspect 2 months postoperation, with complete recovery of the mucosa and normal shape of the alveolar process

**Fig. 9:**
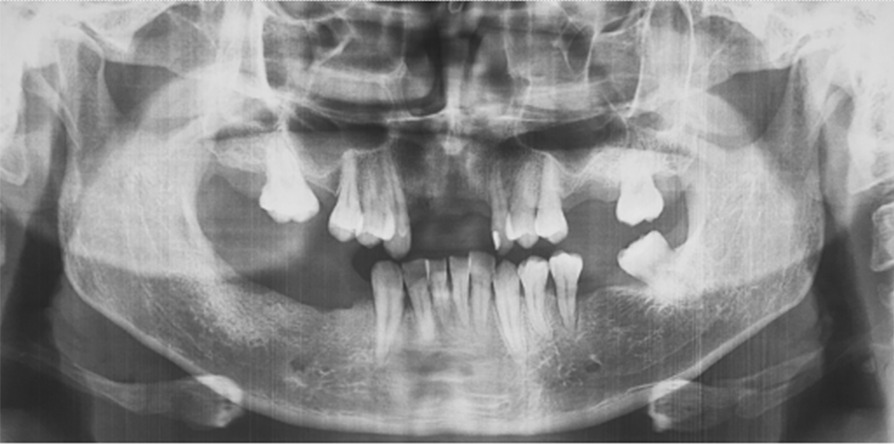
Postoperative panoramic radiograph of the patient, revealing both sizes of the mandible with symmetric shape, proportion, and density

Since the tumor presented both clinical and radiographic features of a benign lesion, the patient underwent an excisional biopsy, with complete removal of the mass and an osteoplasty of the mandible. The access was intraoral, with an incision directly over the lesion, and divulsion of the mucoperiosteal tissues, preserving their integrity for suture later (Fig. 5). After the complete exposure of the lesion, surgical drills were used to mark the limits of excision, and the mass was removed in two pieces, with the use of a chisel and hammer. An oval drill was used to perform an osteoplasty of the jaw, recovering its original shape and thickness (Fig. 6). After surgical resection, the mucosa flaps had their wedges trimmed, to obtain straight margins that were sutured with 3–0 resorbable thread. The surgical piece was a white, oval, bony fragment, with a regular surface of approximately 4.2 × 4.8 × 2.5 cm (Fig. 7). The postoperative course was uneventful, except for discrete dehiscence of the suture 5 days after the procedure, which was spontaneously healed with chlorhexidine mouthwash on clinical follow-up for the next 7 days (Fig. 8). The radiographic aspect of the jaw, 14 days after surgery showing regular shape and dimensions (Fig. 9).

Histopathological analysis revealed mature adipose tissue, permeated by viable compact bone lamellae, consisting of medullary tissue, delimited by a thin and well-vascularized lamellar cortical bone. Thin and congested blood vessels were noted throughout the whole sample.

## Discussion

Osteomas are benign neoplasms that continue to grow slowly and painlessly over time and are characterized by the proliferation of compact or cancellous bone. The tumor presents as three different types: central osteoma originates from the endosteum, whereas the peripheral type derives from the periosteum and the extraskeletal develops in soft tissues [1, 4, 8].

The development of osteomas is usually asymptomatic and is only detected as a radiographic finding. It does not cause bone reabsorption or destruction, allowing conservative treatment for small lesions [5, 10, 16]. However, in some cases, particularly peripherals, the tumors can achieve great proportions, causing facial asymmetry and loss of function [2, 8]. In such cases, the surgical intervention represents a means of cure, with very few recurrences reported [2, 5, 15]. In our case, despite only 6 months of follow-up, we believe that no recurrence will occur as total resection of the lesion was performed. Central osteomas take longer to present clinical manifestations because considerable growth must occur before expanding cortical limits [2].

Histologically, osteomas can be compact, cancellous, or mixed. Compact osteoma has been defined as hard, dense bone with minimal marrow spaces and occasional Haversian canals, while the cancellous (or spongious) lesion, as in our case, contains trabeculae of bone, fibrofatty-marrow-enclosing osteoblasts, and architecture resembling mature bone [4, 10, 16, 17]. The histology of a mixed osteoma would consist of an association of both [11].

Peripheral osteomas manifest themselves as unilateral, well-circumscribed swelling, resembling the bone in origin, usually with a pedunculated shape and narrow contact area between the lesion and the compact bone [12, 14].

Differential diagnosis of osteoma includes exostosis, which is histologically identical [9], but the clinical features of this case exclude this possibility. Exostoses are usually symmetrical and initiate their growth in the prepubescent phase of life, ceasing to grow once the individual reaches maturity [2, 6, 12]. In the present case, the tumor not only began to grow many years after adolescence but also kept growing for the last 8 years. The clinical, radiographic, and microscopical features of the case described in this article are compatible with the diagnosis of the cancellous type of peripheral osteoma of the mandible.

The pathogenesis of osteoma is still unclear, but a few hypotheses have been proposed and refuted. The theory that the lesion is developmental and caused by congenital anomalies seems unlikely, since most cases were observed in adult patients, and a genetic condition should manifest itself in the formative period of growth [5, 8, 13, 14]. Another proposal, which is no longer held, is that an osteoma is a neoplastic proliferation, caused by chronic inflammation, but if that were the case, the growth of all osteomas should be fast and unlimited [5]. Contrary to that theory, osteomas present a limited potential for growth and slow rate [14]. It is also important to note that the recurrence of peripheral osteomas after their excision is extremely rare [3], and a malignant transformation has never been reported [5, 8].

The reactive theory has been accepted as the best explanation for the pathogenesis of peripheral osteoma, consisting of a combination of trauma and muscle activity. Trauma would cause subperiosteal bleeding or edema, and the muscle traction would locally elevate the periosteum, initiating an osteogenic reaction. The initiating trauma could be minor, being easily forgotten by the patient, and the continuous muscle traction in the area could perpetuate the reaction, causing the growth of the tumor [1, 13, 14, 17].

In this case, particularly, the development of the lesion was incisively traced back by the patient to after a tooth extraction, thus corroborating the theory that it can be a reactive mechanism triggered even by small traumas, which the patient can forget over the years [4]. There are only a few reported cases associating trauma to the origin of peripheral osteoma in the mandible, most of them in the mandibular angle, where the bone suffers traction from the masseter muscle [9, 13, 16]. According to Kaplan [14], the analysis of the precise location of peripheral osteomas in mandibles revealed that almost all cases occurred in proximity to areas of muscle attachment, suggesting that muscle traction may play a role in their development. In this case, however, the tumor developed on the alveolar surface of the mandible, where no muscle forces are present.

## Conclusion

Although the etiopathogenesis of peripheral osteoma is not completely elucidated, the present case strongly suggests an association between a trauma (dental extraction) and the development of the tumor in the mandible, with a favorable evolution after surgical excision.

## Data Availability

Not applicable.
